# Bariatric surgery as a treatment for pseudotumor *cerebri:* case study and narrative review of the literature: Cirurgia bariátrica como tratamento para pseudotumor *cerebri*: estudo de caso e revisão narrativa de literatura

**DOI:** 10.1590/1516-3180.2016.0305060117

**Published:** 2017-05-15

**Authors:** Everton Cazzo, Martinho Antonio Gestic, Murillo Pimentel Utrini, Felipe David Mendonça Chaim, Fábio Henrique Mendonça Chaim, Elaine Cristina Cândido, Luciana Bueno da Silveira Jarolavsky, Ana Maria Neder de Almeida, José Carlos Pareja, Elinton Adami Chaim

**Affiliations:** I MD, MSc, PhD. Assistant Professor, Department of Surgery, Faculdade de Ciências Médicas da Universidade Estadual de Campinas (FCM-UNICAMP), Campinas (SP), Brazil.; II MD, MSc. Assistant Lecturer, Department of Surgery, Faculdade de Ciências Médicas da Universidade Estadual de Campinas (FCM-UNICAMP), Campinas (SP), Brazil.; III MD. Assistant Lecturer, Department of Surgery, Faculdade de Ciências Médicas da Universidade Estadual de Campinas (FCM-UNICAMP), Campinas (SP), Brazil.; IV MD, MSc. Assistant Physician, Department of Surgery, Faculdade de Ciências Médicas da Universidade Estadual de Campinas (FCM-UNICAMP), Campinas (SP), Brazil.; V MD. Resident Physician, Department of Surgery, Faculdade de Ciências Médicas da Universidade Estadual de Campinas (FCM-UNICAMP), Campinas (SP), Brazil.; VI BSc, MSc. Assistant Nurse, Bariatric Surgery Outpatient Clinic, Hospital de Clínicas da Universidade Estadual de Campinas (HC-UNICAMP), Campinas (SP), Brazil.; VII BSc. Head Nurse, Bariatric Surgery Outpatient Clinic, Hospital de Clínicas da Universidade Estadual de Campinas (HC-UNICAMP), Campinas (SP), Brazil.; VIII BSc. Attending Psychologist, Bariatric Surgery Outpatient Clinic, Hospital de Clínicas da Universidade Estadual de Campinas (HC-UNICAMP), Campinas (SP), Brazil.; IX MD, PhD. Associate Professor, Department of Surgery, Faculdade de Ciências Médicas da Universidade Estadual de Campinas (FCM-UNICAMP), Campinas (SP), Brazil.; X MD, MSc, PhD. Full Professor. Department of Surgery, Faculdade de Ciências Médicas da Universidade Estadual de Campinas (FCM-UNICAMP), Campinas (SP), Brazil.

**Keywords:** Pseudotumor cerebri, Obesity, Bariatric surgery, Gastric bypass, Intracranial hypertension

## Abstract

**CONTEXT:**

Pseudotumor *cerebri* occurs when there is an increase in intracranial pressure without an underlying cause, usually leading to loss of vision. It is most commonly observed in obese women of child-bearing age.

**CASE REPORT:**

A 46-year-old woman presented at our service with idiopathic intracranial hypertension that had been diagnosed two years earlier, which had led to chronic refractory headache and an estimated 30% loss of visual acuity, associated with bilateral papilledema. She presented partial improvement of the headache with acetazolamide, but the visual loss persisted. Her intracranial pressure was 34 cmH_2_O. She presented a body mass index of 39.5 kg/m^2^, also associated with high blood pressure. Computed tomography of the cranium with endovenous contrast did not show any abnormalities. She underwent Roux-en-Y gastric bypass with uneventful postoperative evolution. One month following surgery, she presented a 24% excess weight loss. An ophthalmological examination revealed absence of visual loss and remission of the papilledema. There were no new episodes of headache following the surgery. There was also complete resolution of high blood pressure. The intracranial pressure decreased to 24 cmH_2_O, six months after the surgery.

**CONCLUSION:**

Although the condition is usually associated with obesity, there are few reports of bariatric surgery among individuals with pseudotumor *cerebri*. In cases studied previously, there was high prevalence of resolution or improvement of the disease following bariatric surgery. There is no consensus regarding which technique is preferable. Thus, further research is necessary in order to establish a specific algorithm.

## INTRODUCTION

Pseudotumor *cerebri* (PC), also known as benign or idiopathic intracranial hypertension (IIH), is a disorder of elevated intracranial pressure (ICP) that primarily affects obese women of childbearing age, but can also affect non-obese adults and children.^1^^,^^2^ IIH occurs predominantly in women, especially in the age range from 20 to 45, who are four to eight times more likely than men to be affected.^1^^,^^2^ The incidence is approximately 2/100,000 and, given the global obesity epidemic, is likely to rise further.^3^ According to the Dandy criteria, as revised by Friedman and Jacobson, IIH is diagnosed when six criteria are fulfilled:

1. suggestive symptoms or cranial hypertension are present;
2. suggestive signs of cranial hypertension are present;
3. normal cerebrospinal fluid composition;
4. elevation of lumbar puncture opening pressure (> 20 cmH_2_O in lean and > 25 cmH_2_O in obese individuals);
5. no abnormalities on computed tomography or magnetic resonance scans; and
6. no other identifiable cause of intracranial pressure.

Papilledema is typically present.^2^^,^^3^^,^^4^^,^^5^ The underlying pathophysiological mechanisms that lead to this disease remain unknown, and the best-accepted theories are that the change in cranial pressure is linked to increased abdominal pressure, hormonal changes and unrecognized disorders in the cerebral venous system and in relation to cerebrospinal fluid resorption.^4^^,^^5^

Headache is the most common symptom of PC and is present in over 90% of these patients.^6^ Up to 86% develop some degree of visual impairment, which may be severe and even blinding in 10%.^7^

## CASE REPORT

**Figure 1:**
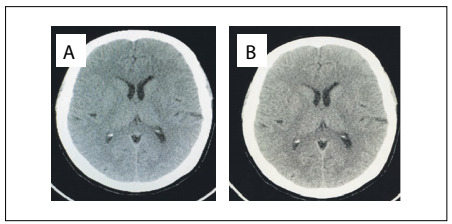
Computed tomography scans: 1A) preoperative; 1B) postoperative.

A 46-year-old woman presented at our service with previously diagnosed IIH that had been diagnosed two years earlier, which had led to chronic refractory headache and an estimated 30% loss of visual acuity, associated with bilateral papilledema. She presented partial improvement of the headache with acetazolamide, but the visual loss persisted. The intracranial pressure, measured by means of lumbar puncture, was 34 cmH_2_O. Biochemical, cytological and microbiological assessments on the cerebrospinal fluid did not reveal any abnormalities. Contrast-enhanced computed tomography did not show any abnormalities either (Figure 1a). She has been obese for 20 years and, on admission, presented a body mass index (BMI) of 39.5 kg/m^2^, also associated with high blood pressure that was controlled through use of enalapril maleate.

She underwent Roux-en-Y gastric bypass with uneventful postoperative evolution. One month after surgery, the patient reported a slight reduction in the frequency of her episodes of headache and improvement of her visual impairment; there was also complete resolution of her high blood pressure. She had lost 24% of her excess weight by then. Use of acetazolamide and enalapril maleate was discontinued at this point.

Six months after the surgery, she presented a 55% loss of excess weight, such that her BMI was 31.5 kg/m^2^. An ophthalmological examination revealed absence of visual loss and remission of the papilledema. The patient reported that there had been a more significant reduction in the frequency and intensity of the episodes of headache since her previous postoperative report. Lumbar puncture was performed again and the opening pressure was found to have decreased to 24 cmH_2_O. There were no abnormalities in the biochemical, cytological and microbiological assessments on cerebrospinal fluid. Table 1 details the characteristics of the cerebrospinal fluid before and six months after surgery. Contrast-enhanced computed tomography showed that no abnormalities were present (Figure 1b).

**Table 1.** General cerebrospinal fluid characteristics before and after surgery in a patient with pseudotumor *cerebri* who underwent bariatric surgery

	Preoperative	Postoperative
Protein (mg/dl)	26	23
Glucose (mg/dl)	62	55
pH	7.3	7.31
Red blood cells (cells/mm^3^)	0	0
White blood cells (cells/mm^3^)	2	1
Gram stain	Negative	Negative
Culture	Negative	Negative

## DISCUSSION

There are several treatment options for IIH. They may aim towards providing headache prophylaxis through using propranolol, amitriptyline and topiramate, or towards alleviation of intracranial pressure and optic symptoms through using diuretics such as acetazolamide and furosemide. In refractory cases, use of corticosteroids may be warranted. There is also the possibility of surgical interventions that aim to decrease the intracranial pressure (through lumbar or ventriculoperitoneal shunts) or alleviate the visual damage (through optic nerve sheath fenestration). The outcomes previously reported from these surgical procedures have been mixed and somewhat frustrating.^1^^,^^2^^,^^3^^,^^4^^,^^5^^,^^6^^,^^7^

Although usually associated with obesity, there are few reports of bariatric surgery among individuals with PC. A review of the literature was conducted through an online search for the MeSH terms “pseudotumor cerebri” and “bariatric surgery” in MEDLINE (via PubMed) and LILACS (via BVS) (Table 2).

**Table 2.** Database search results for bariatric surgery among individuals with pseudotumor *cerebri*, on November 19, 2016

Electronic databases	Search strategies	Results
MEDLINE (PubMed)	((Pseudotumor cerebri) OR (Intracranial hypertension)) AND (Bariatric surgery)	3 systematic reviews 1 retrospective cohort study 2 case series 8 case reports
LILACS (BVS)	(((Pseudotumor cerebri) OR (Pseudotumor cerebral) OR (Seudotumor cerebral)) OR ((Intracranial hypertension) OR (Hipertensão Intracraniana) OR (Hipertensión Intracraneal))) AND ((Bariatric surgery) OR (Cirugia Bariátrica) OR (Cirurgia Bariátrica))	2 systematic reviews 1 retrospective cohort study 1 case series 4 case reports

**Figure 2.**
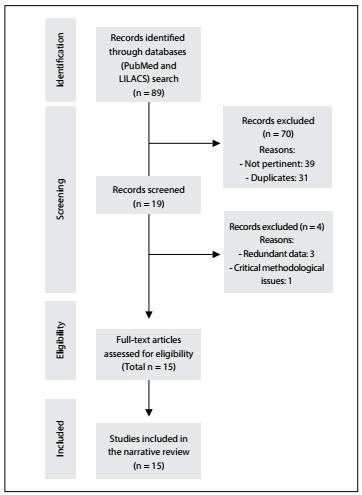
Flow diagram of the narrative review of the literature.

There was significant overlap between the databases. After careful analysis, we selected three systematic reviews, one retrospective cohort study, two case series and nine case reports that evaluated bariatric surgery in individuals with PC, or that compared bariatric surgery with other treatment regimens. Table 3 ^8^^,^^9^^,^^10^^,^^11^^,^^12^^,^^13^^,^^14^^,^^15^^,^^16^^,^^17^^,^^18^^,^^19^^,^^20^^,^^21^^,^^22^ summarizes the main articles selected and their respective levels of evidence according to the Oxford classification, and the results observed. Figure 2 is a flow diagram showing the literature search and selection of articles.

**Table 3.** Main studies on bariatric surgery among individuals with pseudotumor *cerebri*

Study	Methods	N	Level of evidence	Treatment option	Main results
Kalyvas et al.^8^	Systematic review of case series and case reports	728	3a	341: optic nerve sheath fenestration 128: lumboperitoneal shunting 72: ventriculoperitoneal shunting 155: venous sinus stenting 32: bariatric surgery (29: gastric bypass; 2: gastroplasty; 1: gastric banding)	The studies were heterogeneous. No type of surgery proved to be clearly superior. Bariatric surgery presented a higher success rate among obese individuals but also a higher morbidity rate.
Roth et al.^9^	Case series	13 (6: shunting and bariatric surgery; 7: only shunting)	4	Bariatric surgery in individuals who previously underwent shunting procedures vs. only shunting (4: sleeve gastrectomy; 2: gastric banding)	Bariatric surgery is less effective and may lead to over-drainage symptoms in individuals who previously underwent shunting
Handley et al.^10^	Systematic review	65	3a	Bariatric surgery (48: gastric bypass; 6: gastric banding; 4: gastroplasty; 5: sleeve gastrectomy)	Overall improvement in 92% of the individuals; mean 18.9 cmH_2_O decrease in lumbar puncture pressure
Levin et al.^11^	Case report	1	4	Gastric bypass	Improvement of headache and reversal of papilledema
Egan et al.^12^	Case series	4	4	Gastric banding	Total resolution or significant improvement of headache (mean improvement in pain score of 76.3/100 (range 55-95) on an analogue pain scale)
Fridley et al.^13^	Systematic review	62	3a	Bariatric surgery (55: gastric bypass; 4: gastroplasty; 3: gastric banding)	Resolution of headache in 92% of the individuals; mean 25.4 cmH_2_O decrease in CSF pressure
Williams et al.^14^	Case report	1	4	Gastric banding	Complete resolution of headache and visual loss
Stangherlin et al.^15^	Case report	1	4	Gastric banding	Complete resolution of headache and CSF rhinorrhea
Leslie et al.^16^	Case report	1	4	Gastric bypass	Complete resolution of visual loss
Chandra et al.^17^	Case report	1	4	Gastric bypass	Complete resolution of visual loss
Soto et al.^18^	Case report	1	4	Gastric bypass	Complete resolution of headache, visual loss and dizziness
Lazcano-Herrera et al.^19^	Case report	1	4	Modified jejunocolic bypass	Complete resolution of headache and visual loss
Nadkarni et al.^20^	Case report	2	4	1: gastric bypass 1: gastric stapling	Complete resolution of headache and visual loss
Fontes et al.^21^	Case report	1	4	Gastric bypass	Complete resolution of headache and visual loss
Sugerman et al.^22^	Retrospective cohort	24	2b	23: gastric bypass 1: gastric banding	Resolution of visual loss in 100% and headache and tinnitus in 94.7% of the individuals

N = number of individuals; CSF = cerebrospinal fluid. Levels of evidence according to the Oxford classification - 1a = Systematic reviews (with homogeneity) of randomized controlled trials; 1b = Individual randomized controlled trials (with narrow confidence interval); 1c =: “All or none” randomized controlled trials; 2a = Systematic reviews (with homogeneity) of cohort studies; 2b = Individual cohort study or low-quality randomized controlled trials (e.g. < 80% follow-up); 2c = “Outcomes” research; ecological studies; 3a = Systematic review (with homogeneity) of case-control studies; 3b = Individual case-control study; 4 = Case series (and poor-quality cohort and case-control studies); 5 = Expert opinion without explicit critical appraisal, or based on physiology, bench research or “first principles”.

Although two systematic reviews^10^^,^^13^ were found, the majority of the studies that evaluated bariatric surgery as a treatment for IIH were case reports and case series. Thus, the quality of most of the evidence available so far is poor. The present report is only the second published case on pseudotumor *cerebri* that was treated by means of bariatric surgery in Brazil. The first case was reported by Fontes et al.,^21^ who observed that complete resolution of PC-related symptoms was achieved in a 37-year-old female after RYGB. Nadkarni et al.^20^ reported on two cases of obese women, both aged 42 years, who underwent gastric bypass and gastric stapling. Complete resolution of PC was observed at the one-year reevaluation. Soto et al.^18^ described the case of a 30-year-old woman who presented complete resolution of headache, visual impairment and dizziness three months after undergoing laparoscopic RYGB. Levin et al.^11^ reported the case of a 29-year-old obese woman with PC who presented dramatic improvement of headache four months after laparoscopic RYGB, and maintained this improvement one year after surgery. The case reported in our study also presented early improvement in PC-related symptoms, similar to the previously published evidence.

Roth et al.^9^ compared individuals who underwent ventricular shunt surgery alone or in association with bariatric surgery. Their study revealed that, among shunted patients, bariatric surgery might not lead to resolution of PC-related symptoms and that these patients might remain shunt-dependent.

The systematic review conducted by Fridley et al.^13^ identified a total of 62 individuals with PC who underwent bariatric surgery. They observed that the resolution rate for PC-related symptoms following bariatric surgery was 92%, with an average postoperative pressure decrease of 25.4 cmH_2_O. These authors concluded that the class IV evidence published up to the time of their study suggested that bariatric surgery might be an effective treatment for PC among obese patients, but that prospective, controlled studies would be necessary for better elucidation of its role. A more recent systematic review by Handley et al.,^10^ which enrolled 65 individuals, showed that there was an overall improvement in PC symptoms after bariatric surgery, in 60 of the 65 patients observed (92%). The postoperative lumbar puncture opening pressure was shown to decrease by an average of 189 mmH_2_O in the patients for whom records of this pressure were available. A comprehensive systematic review conducted by Kalyvas et al.,^8^ which also included studies that evaluated other treatment options, evaluated 32 individuals who underwent bariatric surgery. They observed that papilledema resolved in all patients and that headache improvement was documented in 96% of the patients, with no deterioration in any of the patients. However, these authors also emphasized that there was a higher degree of morbidity in the bariatric surgery group, compared with the other treatment regimens evaluated in other studies.

Despite the growing evidence of high rates of improvement and even resolution of PC achieved by means of bariatric surgery, the low quality of most of the available evidence means that no ultimate conclusions can be reached. Nonetheless, there is an increasing perception that obese individuals with increased intracranial hypertension may significantly benefit from bariatric surgery. Hence, individuals who fulfill the current indications for bariatric surgery should at least be offered this type of treatment.

## CONCLUSION

Bariatric surgery led to early improvement in the PC-related symptoms in this report, and this was comparable with evidence published previously.
